# Systematic evaluation of velocity‐selective arterial spin labeling settings for placental perfusion measurement

**DOI:** 10.1002/mrm.28240

**Published:** 2020-03-06

**Authors:** Anita A. Harteveld, Jana Hutter, Suzanne L. Franklin, Laurence H. Jackson, Mary Rutherford, Joseph V. Hajnal, Matthias J. P. van Osch, Clemens Bos, Enrico De Vita

**Affiliations:** ^1^ Department of Radiology University Medical Center Utrecht Utrecht University Utrecht the Netherlands; ^2^ Biomedical Engineering School of Imaging Sciences and Biomedical Engineering King's College London London United Kingdom; ^3^ C.J. Gorter Center for high field MRI, Department of Radiology Leiden University Medical Center Leiden the Netherlands

**Keywords:** arterial spin labeling (ASL), magnetic resonance imaging, perfusion, placental function, velocity‐selective arterial spin labeling

## Abstract

**Purpose:**

Placental function is key for successful human pregnancies. Perfusion may be a sensitive marker for the in vivo assessment of placental function. Arterial spin labeling (ASL) MRI enables noninvasive measurement of tissue perfusion and it was recently suggested that ASL with velocity‐selective (VS) labeling could be advantageous in the placenta. We systematically evaluated essential VS‐ASL sequence parameters to determine optimal settings for efficient placental perfusion measurements.

**Methods:**

Eleven pregnant women were scanned at 3T using VS‐ASL with 2D multislice echo planar imaging (EPI)‐readout. One reference VS‐ASL scan was acquired in all subjects; within subgroups the following parameters were systematically varied: cutoff velocity, velocity encoding direction, and inflow time. Visual evaluation and region of interest analyses were performed to compare perfusion signal differences between acquisitions.

**Results:**

In all subjects, a perfusion pattern with clear hyperintense focal regions was observed. Perfusion signal decreased with inflow time and cutoff velocity. Subject‐specific dependence on velocity encoding direction was observed. High temporal signal‐to‐noise ratios with high contrast on the perfusion images between the hyperintense regions and placental tissue were seen at ~1.6 cm/s cutoff velocity and ~1000 ms inflow time. Evaluation of measurements at multiple inflow times revealed differences in blood flow dynamics between placental regions.

**Conclusion:**

Placental perfusion measurements are feasible at 3T using VS‐ASL with 2D multislice EPI‐readout. A clear dependence of perfusion signal on VS labeling parameters and inflow time was demonstrated. Whereas multiple parameter combinations may advance the interpretation of placental circulation dynamics, this study provides a basis to select an effective set of parameters for the observation of placenta perfusion natural history and its potential pathological changes.

## 1 INTRODUCTION

The human placenta is a vital organ during pregnancy. It links the developing fetus via the umbilical cord to the blood supply of the mother to allow gas exchange, uptake of nutrients, and removal of waste products. Therefore this organ plays a crucial role for healthy fetal development. Major pregnancy complications have been linked with placental dysfunction, such as fetal growth restriction, preeclampsia, and preterm birth, all associated with neonatal mortality, morbidity, and adverse long‐term outcome.^1^, ^2^, ^3^, ^4^, ^5^, ^6^

The placenta is a highly vascularized organ, perfused by both the maternal and fetal circulations, consequently, vascular/hypoxic abnormalities are often at the core of placental dysfunction.^7^, ^8^, ^9^ Perfusion may thus be a sensitive marker for the assessment of placental function. Clinical diagnostic tools to assess placental function during gestation are currently limited to indirect measurement of blood flow pulsatility index in the uterine or umbilical arteries with Doppler ultrasound.^10^, ^11^, ^12^ In recent years, interest for in vivo placental MRI has increased due to advances in this field, bringing clinical placental MRI within reach. MRI offers the potential for direct noninvasive assessment of the entire placenta, and for combined morphological and functional assessment across gestation.^13^ Arterial spin labeling (ASL) MRI enables noninvasive measurement of tissue perfusion by employing magnetically labeled blood water as an endogenous tracer.^14^ These features make ASL an attractive method for placental application, and a number of publications have demonstrated its value in humans.^15^, ^16^, ^17^, ^18^, ^19^, ^20^, ^21^

Commonly used spatially selective labeling approaches (ie, pulsed and pseudocontinuous ASL)^22^ have been successfully applied in organs like the brain^23^ and kidneys,^24^ where the geometry of the vasculature and blood supply is well known and very similar across individuals, so that effective labeling locations can be consistently prescribed. The placenta, with its highly variable shape, location, and its double input of maternal and fetal blood is very different: downstream of the descending aorta, the vascular network of uterine, ovarian, and spiral arteries supplying the placenta with maternal blood is complex and highly variable across individuals; the vasculature supplying the placenta with fetal blood via the tortuous umbilical arteries, chorionic arteries, and villous trees is also extremely intricate. As a result, for the placenta, planning of a spatially selective labeling slab with respect to the feeding arteries can be challenging to preserve a controllable upstream location and still create label close enough to avoid excessive label relaxation.^20^, ^21^ Hence, a spatially nonselective labeling method that creates a label directly within the imaging region, such as velocity‐selective (VS) ASL,^25^ may be better suited for measuring placental perfusion. An additional benefit of this labeling approach is that no prior knowledge regarding the arteries feeding the placenta is required to perform labeling.

Two recent studies from the same research lab, have proposed VS‐ASL for placental perfusion imaging,^19^, ^21^ but without exploring optimal sequence parameters. In the brain, where VS‐ASL methods have been recently established, a cutoff velocity of ~2 cm/s is most frequently used, based upon the velocity in cortical penetrating arterioles, and the inflow time is usually set around the *T*_1_ of blood (eg, 1600 ms at 3T).^25^, ^26^, ^27^ The cerebral VS‐ASL signal appears to be relatively insensitive to the velocity encoding direction for cutoff velocities below ~4 cm/s. Typically a second VS module is used before the image readout to reduce venous signal contributions and to allow quantification.

Previously mentioned placental VS‐ASL studies were performed at 1.5T and employed similar settings as used in brain applications. However, such parameter choice may not be ideal for the placenta. The blood content in the placenta is over an order of magnitude higher than in the brain,^28^ and blood flow regimes to and within the intervillous space (IVS) are also completely different from the known perfusion regimes in the cerebral cortex. With this in mind, it is important to seek parameter settings that can yield high sensitivity to the slow flowing blood in the placenta and provide an effective scan to assess placental perfusion, which can be easily planned and is tolerable for pregnant women.

Therefore, in this study, we systematically evaluated essential VS‐ASL sequence parameters (cutoff velocity, velocity encoding direction, inflow time, and the addition of a second VS module before image acquisition) to determine optimal settings for placental perfusion measurements. This study also adds to the evidence that VS‐ASL in the placenta is feasible and, thereby, provides repeatability evidence for the two previous placental VS‐ASL studies.^19^, ^21^ This is especially important, since it is challenging to perform lengthy technical development studies in pregnant women.

## 2 METHODS

### 2.1 Study population

This prospective study was approved by the local institutional review board; all subjects provided written informed consent. Study participants were recruited from St Thomas’ Hospital, London, between July 2018 and June 2019. Inclusion criteria included pregnant volunteers, at least 18 years of age, body mass index <30, with singleton pregnancies, gestational age (GA) in the second or third trimester, anteriorly located placentas (which was determined from prior routine ultrasound examinations), and without contraindications for MR imaging. The criteria limiting the placental location to anterior was chosen to make the study population more homogeneous and to limit variability in measurements resulting from differences in placenta location.^19^

### 2.2 MR Imaging

Imaging was performed on a 3T MR system (Achieva, Philips, Best, the Netherlands, software release 3.2.2) equipped with a 32‐channel cardiac coil. All subjects were scanned under free‐breathing conditions in supine position.^29^ Subjects were all scanned in the same pose to reduce the possible influence of subject pose and receiver coil placement on the measured perfusion signal.^19^ They were monitored using blood pressure measurements at 10‐min intervals and received continuous heart rate and oxygen saturation assessment during the scan session. Prior to the ASL data acquisition, a 2D *T*_2_‐weighted turbo spin echo sequence was acquired to enable correct planning of subsequent acquisitions, and a static magnetic field (B_0_) map was obtained covering the whole uterus to perform image‐based shimming.^30^ For fetal safety, all scans underwent acoustic testing prior to in vivo scanning with an MR‐compatible fiber optic microphone (Optimic 1155, Optoacoustics Ltd, Israel); the maximum sound pressure level was set at 105 dB(A).

#### 2.2.1 ASL sequence

The ASL sequence consisted of a VS labeling module, a post‐labeling delay (PLD; ie, inflow time) including background suppression (BGS), and a gradient‐echo EPI‐readout. A software patch was used to perform the VS‐ASL sequence.

The VS labeling module was implemented based on the double refocused hyperbolic secant design (DRHS).^25^, ^27^ Motion‐sensitizing gradients in the VS module tag blood by saturating the magnetization of blood that flows faster than a set cutoff velocity, under the assumption of a laminar flow profile. Control and label images were acquired interleaved. Since in the control condition no motion‐sensitizing gradients are applied, the signal difference between control and label images is due to the flowing/tagged spins.

The image readout consisted of a single‐shot gradient‐echo EPI 2D multislice readout with an EPI‐factor of 39, a SENSE‐factor of 2.5, and phase‐encoding set to right‐left. To limit acoustic noise, the EPI‐sequence was constrained by imposing an echo spacing of 0.98 ms (ie, readout frequency of ~510 Hz), shown previously^31^ to minimize acoustic noise on the scanner. Thirteen slices (in a coronal orientation relative to the mother) were acquired with ascending slice acquisition order in basal to chorionic plate direction, covering a 350 × 334 mm^2^ FOV with an in‐plane resolution of 4 × 4 mm^2^, slice thickness of 4 mm, slice gap of 0.4 mm, echo time of 22 ms, and readout time for each slice of 69 ms. Fat‐suppression was achieved by application of the “spectral presaturation with inversion recovery” (SPIR) technique. A coronal slice orientation relative to the mother was chosen to ensure that most of the anteriorly located placenta could be captured within the relatively small imaging stack thickness (57 mm).

BGS consisted of two adiabatic non‐slice‐selective inversion pulses (hyperbolic‐secant) applied during the PLD. Timings of the inversion pulses were chosen based on preliminary scans and *T*_1_ measurements of the placenta.^32^ We aimed to have the zero crossing of the longitudinal magnetization of the placental tissue occur just before acquisition of the first slice.

Each ASL scan consisted of 26 label‐control pairs for signal averaging. One control image without BGS was added to each ASL scan to be used for normalization of the perfusion‐ weighted images, referred to as the pseudo–proton density image (pseudo‐M_0_).

### 2.3 Experiments

#### 2.3.1 Reference ASL scan

The “reference” ASL scan was defined with settings close to those applied in previous brain^25^, ^27^ and placenta^19^, ^21^ studies; using a cutoff velocity of 1.6 cm/s and PLD of 1600 ms. During the PLD, BGS pulses were applied at 50 and 1160 ms following the VS module. The blood flow direction in the vicinity of the IVS is expected to be in the direction from basal to chorionic plate; a vector sum of the individual directions is likely to have the largest component roughly corresponding to anterior‐posterior (AP) direction for anterior placentas and, therefore, was chosen as the reference velocity encoding direction of the VS module. In the reference sequence only one VS module was applied (referred to as “single VS‐ASL”^27^) to tag all spins flowing faster than the set cutoff velocity, accepting that both arterial and venous blood flow will contribute to the signal. The sequence repeat time, TR, was 3500 ms, resulting in a total acquisition time of 3 min 16 s. The reference ASL scan was acquired in all subjects.

Subsequently for each subject, sequence parameters were systematically varied by adapting one parameter at a time. The following VS‐ASL scan parameter settings were evaluated: cutoff velocity, velocity encoding direction, PLD, and the addition of a second VS module. Except for one subject, the reference ASL scan was always performed first, with the order of the following scans randomized. Table 1 specifies which parameters were studied in each subject.

**Table 1:** Baseline characteristics of the study population included for analysis

Subject	Maternal age (years)	Gestational age at MRI (weeks)	Relevant medical history	Experiments				
				Reference	Velocity encoding direction	Cutoff velocity	PLD	Dual VS‐ASL
1	42.9	33.7	Preeclampsia	✓	✓			
2	31.8	29.6	–	✓		✓		✓
3	33.7	30.0	–	✓	✓			
4	36.4	29.1	–	✓			✓	✓
5	36.7	29.4	–	✓			✓	✓
6	32.6	30.9	–	✓			✓	✓
7	30.6	24.3	–	✓			✓	✓
8	37.7	28.3	–	✓		✓		✓
9	30.0	28.3	–	✓		✓		
10	38.9	35.1	–	✓	✓			

#### 2.3.2 Cutoff velocity

The formula described by Wu & Wong^33^ was used to calculate the cutoff velocity. In the reference ASL scan, a gradient strength of 13 mT/m, gradient duration of 1.2 ms, and labeling module duration of 50 ms was used, corresponding to a cutoff velocity of 1.6 cm/s. To evaluate the influence of cutoff velocity on the perfusion signal, the VS encoding gradient strength was varied from 19, 6, and 3 mT/m to produce cutoff velocities of 0.9, 4.4, and 10.2 cm/s, respectively (*n* = 3 subjects). An overview of the applied VS module settings for each cutoff velocity with the resulting *b*‐values and estimated subtraction error due to diffusion during the VS module are provided in Supporting Information Table S1.

#### 2.3.3 Velocity encoding direction

The direction of the motion‐sensitizing gradients applied during VS labeling can be chosen arbitrarily and determines the flow direction for which labeling is effective. Velocity encoding was tested along each of three orthogonal directions: AP (reference), superior‐inferior (SI), and right‐left (RL) with respect to the maternal position (*n* = 3 subjects).

#### 2.3.4 PLD

The PLD is the time allowed for the labeled blood to flow into the placental tissue. To investigate its influence on the perfusion signal data were acquired with PLDs of 400, 1000, 1600 (reference) and 2200 ms (*n* = 4 subjects). BGS pulse timings were optimized for each PLD. BGS pulses were applied at 50/310, 50/723, and 848/1780 ms for a PLD of 400, 1000, and 2200 ms, respectively.

#### 2.3.5 Addition of a second VS module before image readout

In the reference ASL scan, single VS‐ASL labeling was performed. An additional identical VS module with the same cutoff velocity as the initial VS module can be applied just before the image readout; referred to as “dual VS‐ASL” as in Schmid et al^27^ or “VSASL/QUIPSS II” as in Wong et al.^25^ For this second VS module, motion‐sensitizing gradients are enabled in both label and control conditions to suppress the contribution of signal from spins that are still above the cutoff velocity just before the image readout. Due to the additional radiofrequency‐pulses of the second VS module and the specific absorption rate limit of 2 W/kg, the TR of the sequence needed to be increased to 6400 ms, resulting in a total acquisition time of 5 min 58 s for the dual VS‐ASL scan, for the same number of averages. This dual VS‐ASL scan was added to the scan protocol in six subjects.

### 2.4 Data processing

Motion correction was performed using Advanced Normalization Tools (ANTs)^34^ to align all non‐subtracted ASL image volumes within/between scans acquired in the same subject. For each subject, all ASL image volumes were registered to a common representative space created using an iterative template construction approach, using ANTs’ *antsMultivariateTemplateConstruction* function. Parameters used for the nonlinear image registration were the script defaults, using the nonlinear Symmetric Normalization (SyN) registration algorithm.^35^

After motion correction, further image processing and analysis were performed with MeVisLab (v2.8.2, MeVis Medical Solutions AG, Bremen, Germany). Label‐control images were pairwise subtracted. Next, for each ASL scan outlier rejection was performed by excluding subtraction images containing >20% voxels (within the uterus region) with a value of more than ±1.5 SD from the mean voxel value over all repetitions. Finally, the remaining subtraction images were averaged (ΔM) and normalized.

**FIGURE 1:**
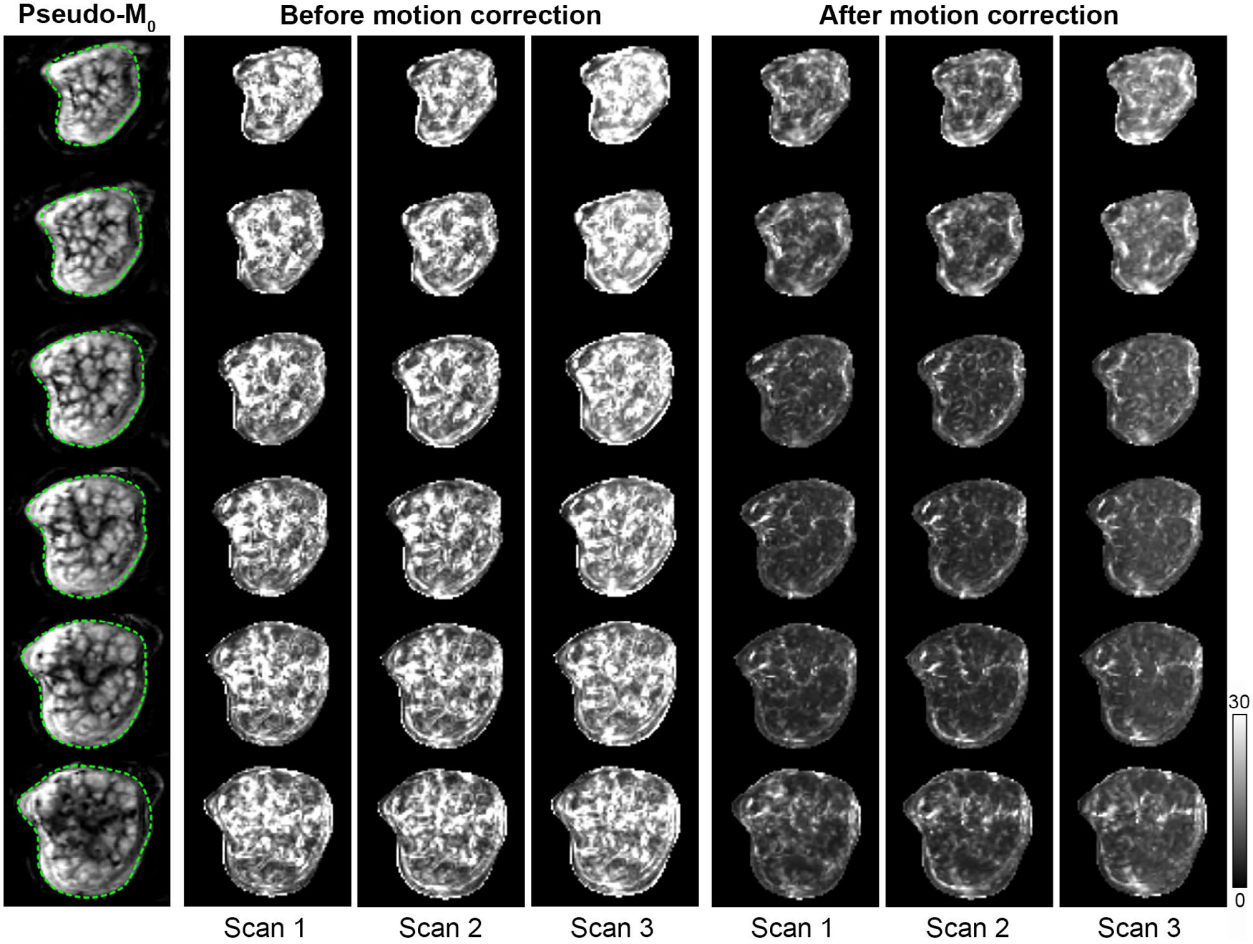
Evaluation of motion correction quality. Images show multiple slices (top to bottom row: anterior‐posterior slice scan order) of the normalized voxel‐wise tSD over all ASL source images of each scan before and after motion correction from one pregnant volunteer (Subject 1 in Table 1). Reduction of the normalized tSD after motion correction can be observed for all scans. The normalized tSD images are shown with equal LUT scaling and were masked around the uterus region (the masked areas are visualized with green dashed lines on the pseudo‐M_0_ images in the first column). The grayscale bar indicates normalized tSD [%]

**FIGURE 2:**
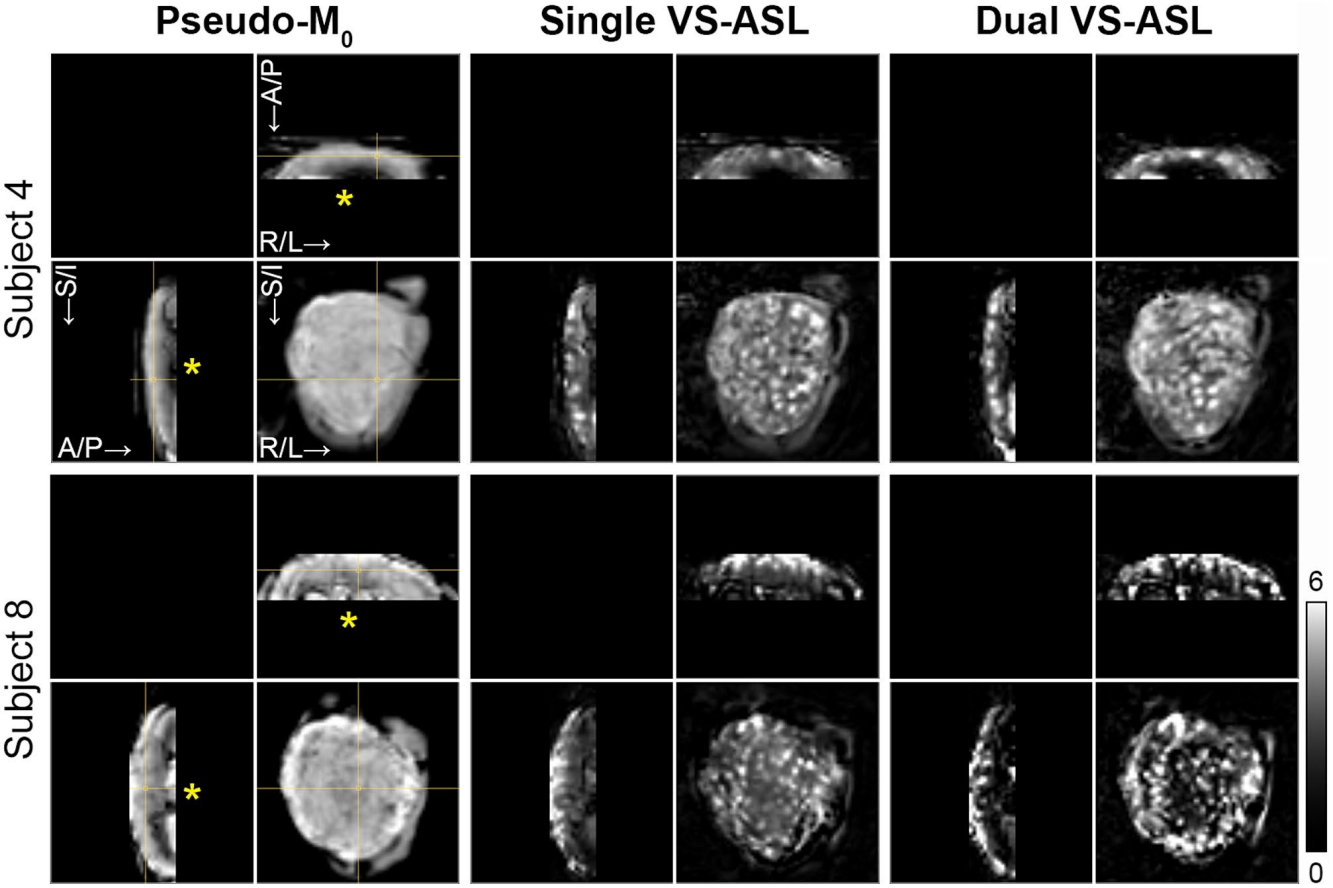
Comparison of single vs. dual VS‐ASL. Placental perfusion images obtained with single (middle column) and dual (right column) VS‐ASL MRI and the corresponding pseudo‐M_0_ images (left column) in two pregnant volunteers (Subject 4 and 8 in Table 1). Orthogonal slices (spatial resolution: 4 × 4 × 4 mm^3^) are shown. The cross‐hair in the pseudo‐M_0_ images indicates the intersection of the slices. The yellow asterisk on the axial and sagittal slices point at the fetal chorionic side of the placenta. PWS images have equal LUT scaling within each subject. The grayscale bar indicates PWS [%]. A/P, anterior‐posterior; R/L, right‐left; S/I, superior‐inferior

The pseudo‐M_0_ images of some subjects showed marked spatial heterogeneity (see Figure 1 vs Figure 2); this was expected from previous placental studies and is linked to deposition of fibrin leading to a clearer delineation of the septa between the lobules with increasing GA.^36^ Since we wanted the M_0_ to represent signal from inflowing blood, a single M_0_ value was used to normalize each ASL scan. Assuming the higher values in the pseudo‐M_0_ image allow an estimate of inflowing blood signal that is independent of the placental development stage, the 80th‐percentile of all voxel values within a region‐of‐interest (ROI) covering the whole placenta (ROI_placenta_; see definition below) was used for the single M_0_ value.

### 2.5 Data analysis

**FIGURE 3:**
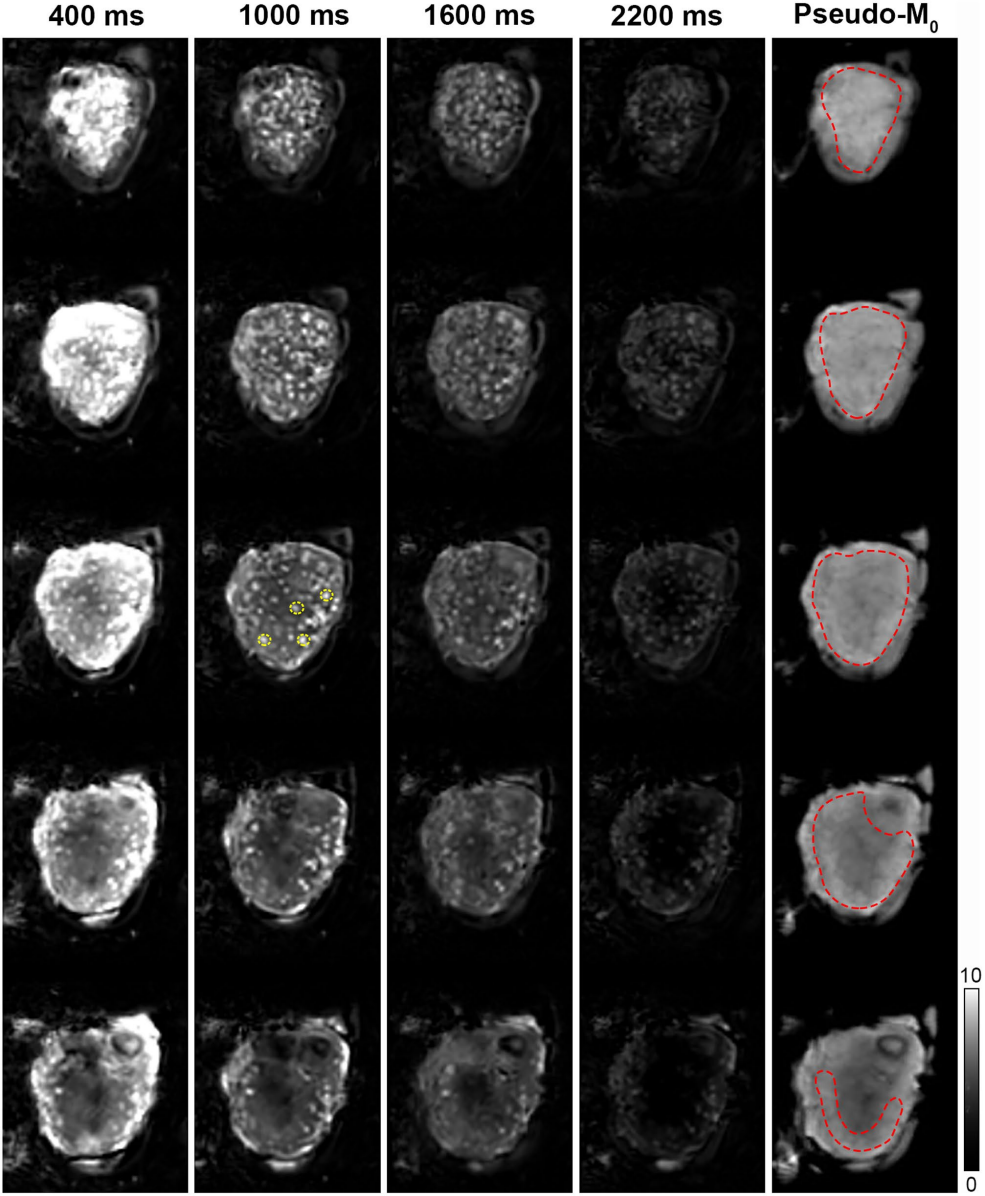
Variation of PLD. Placental perfusion images obtained with single VS‐ASL MRI using four different PLDs (400, 1000, 1600, and 2200 ms) and the corresponding pseudo‐M_0_ images in one pregnant volunteer (Subject 4 in Table 1). Multiple slices (spatial resolution: 4 × 4 × 4 mm^3^) with maternal coronal orientation were acquired in anterior to posterior order (top to bottom in Figure). ROIs were drawn manually in the placenta on the pseudo‐M_0_ images (ROI_placenta_; red dashed lines) and around four hyperintense focal regions identified on the perfusion images (ROI_focal_; yellow dashed lines on third slice of PLD 1000 ms). Perfusion images are shown with equal LUT scaling. The grayscale bar indicates PWS [%]

ROI analyses were performed to quantitatively compare perfusion signal differences between the parameter variations. ROIs were drawn manually by a single observer in the placenta on multiple slices of the pseudo‐M_0_ image (only slices where the organ could be well‐defined were included) to define a whole‐placenta ROI (ROI_placenta_). In addition, ROIs were drawn around four focal hyperintense regions (ROI_focal_) identified on the perfusion‐weighted images (all on the same slice) of the reference ASL scan and were cross‐checked on the other scans of the same subject; see Figure 3. The same ROIs were used for all acquisitions in each subject. For assessment of ASL quality, the relative perfusion‐weighted signal (PWS = (ΔM/M_0_) × 100%) and voxel‐wise temporal signal‐to‐noise ratio (tSNR), averaged over all voxels inside the ROIs, were calculated for each scan. tSNR, obtained from the subtraction images after outlier rejection as a metric for consistency of the perfusion signal over repetitions, was defined as the temporal voxel‐wise mean divided by the temporal voxel‐wise SD (tSD). For evaluation of motion correction quality, the normalized voxel‐wise tSD was calculated over all ASL source images (the acquired label/control images) of each scan before and after motion correction. The tSD values were normalized to the voxel‐wise temporal mean value to compensate for possible global intensity differences introduced by the motion correction algorithm.

Differences between ROI values obtained from the images with different scan parameter settings were analyzed using repeated measures one‐way analysis of variance and Tukey’s post hoc tests for pairwise comparisons. A *P*‐value of < .05 was considered to be statistically significant. Statistical analyses were performed using GraphPad Prism (v8.0.1, GraphPad Software, San Diego, CA, USA).

## 3 RESULTS

Eleven subjects were included. Images were analyzed for 10 subjects (age 35.1 ± 3.9 years; GA 29.9 ± 2.8, range 24.3 – 35.1 weeks; see Table 1); one subject was excluded from analysis due to poor image quality caused by severe fetal motion during acquisition. Nine subjects were healthy, and one subject had preeclampsia.

### 3.1 Registration and outlier rejection

Reduced misalignment within and between ASL scans after motion correction was observed in all subjects. The whole‐placenta normalized voxel‐wise tSD after motion correction for the ASL source images was on average 6.7 ± 3.1% for all scans. Figure 1 illustrates the reduction of normalized tSD after motion correction in Subject 1. On average, 4 (range 2‐7) label‐control pairs were considered outliers and rejected for each VS‐ASL acquisition.

### 3.2 Reference ASL scan

**FIGURE 4:**
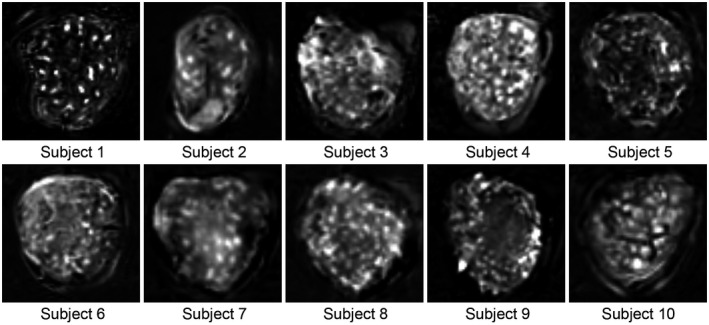
Placental perfusion images obtained with the reference ASL scan in each subject. One slice per subject with maternal coronal orientation is shown (spatial resolution: 4 × 4 × 4 mm^3^). Images were scaled individually

In general, the placental perfusion pattern consisted of distinct small round hyperintense regions spread over the entire placenta located more towards the basal plate side (Supporting Information Figure S1), surrounded by relatively low signal intensity. Representative slices for the reference scan of each subject are shown in Figure 4.

**FIGURE 5:**
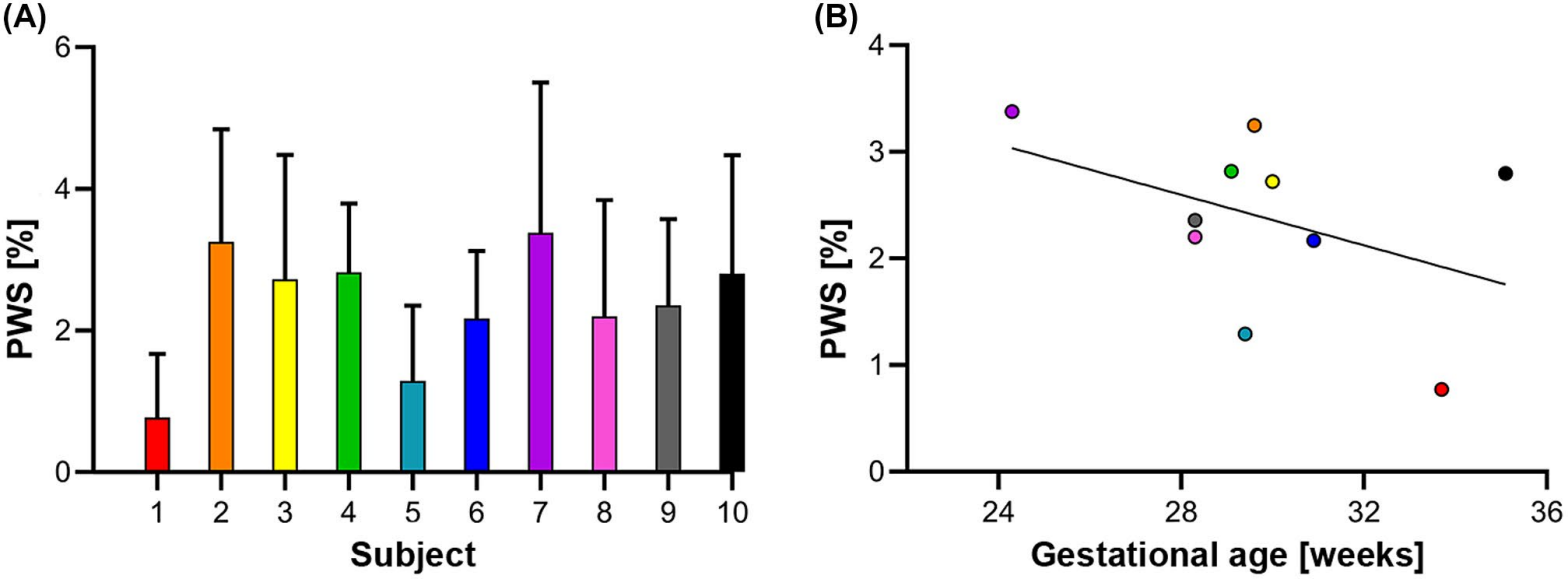
Placental perfusion signal measured with the reference ASL scan. (A) Bar graph of the average PWS in the whole‐placental region (ROI_placenta_) measured in each subject (error bars represent SD). (B) Scatterplot of the relation between PWS and gestational age. Individual subjects are presented with the same color in both graphs

Whole‐placenta PWS for all subjects, and its relation with GA is displayed in Figure 5. PWS was on average 2.4 ± 0.8% (range 0.8‐3.4%) in the whole‐placenta region and 4.3 ± 1.2% (range 2.2‐6.2%) in the focal hyperintense regions. A negative trend was observed between whole‐placenta PWS and GA (Spearman’s rho = −0.35, *P* = .32).

### 3.3 Variation of scan parameter settings

#### 3.3.1 Cutoff velocity dependence

**FIGURE 6:**
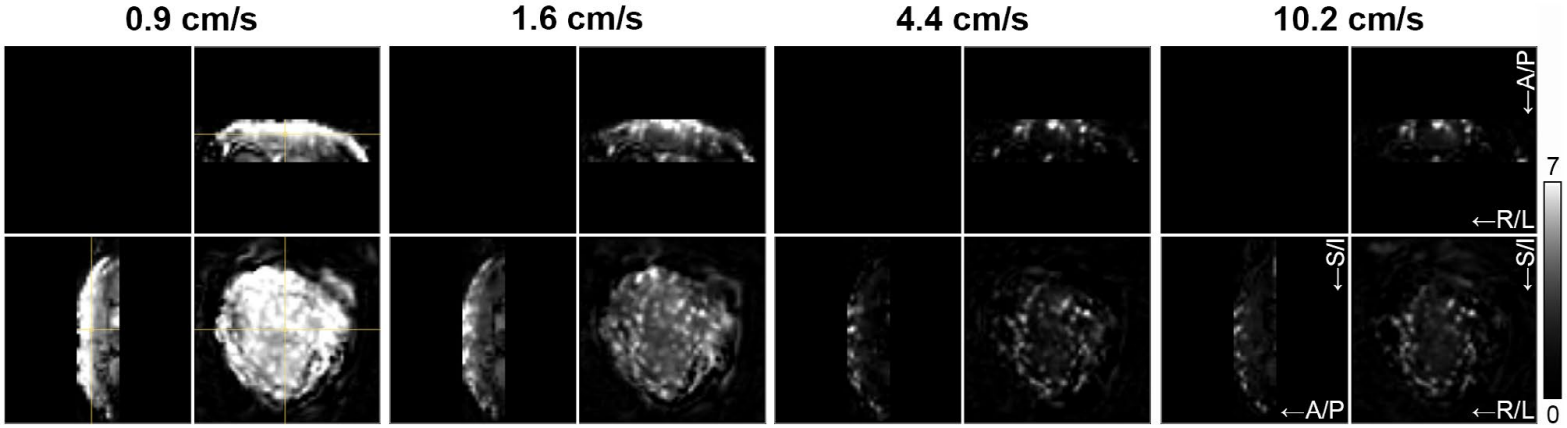
Variation of cutoff velocity. Placental perfusion images obtained with VS‐ASL MRI using four different cutoff velocities (0.9, 1.6, 4.4, and 10.2 cm/s) in a pregnant volunteer (Subject 8 in Table 1). Orthogonal slices (spatial resolution: 4 × 4 × 4 mm^3^) are shown. The cross‐hair in the left images indicates the intersection of the slices. Images have equal LUT scaling. The grayscale bar indicates PWS [%]. A/P, anterior‐posterior; R/L, right‐left; S/I: superior‐inferior

**FIGURE 7:**
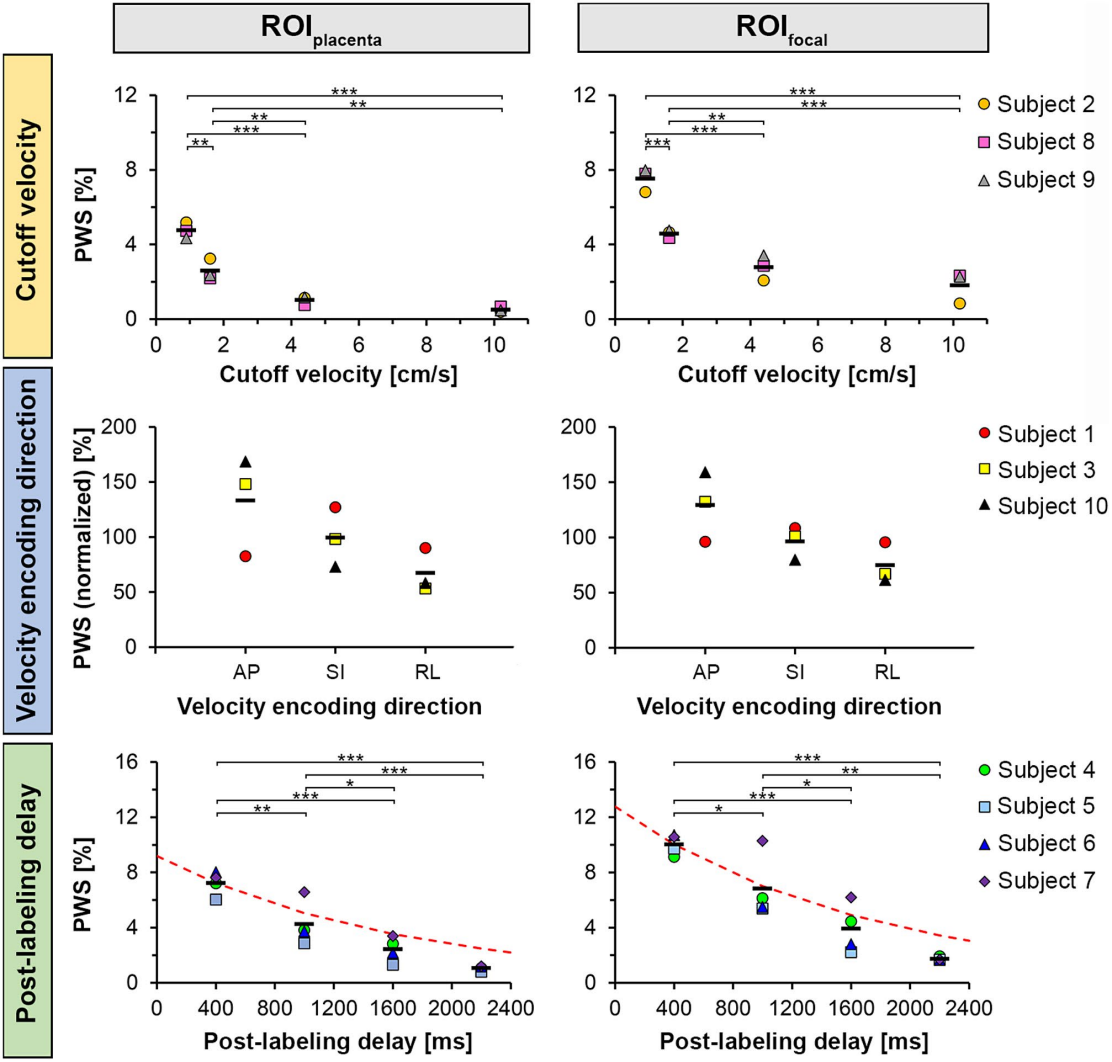
ROI analysis of PWS obtained from VS‐ASL images as a function of cutoff velocity, velocity encoding direction, and PLD. Data points represent the average PWS in the whole‐placenta region (ROI_placenta_; left column) and in focal hyperintense regions (ROI_focal_; right column). Red dotted lines indicate exponential *T*_1_ decay starting from the ROI‐averaged PWS value at a PLD of 400 ms (*T*_1,blood_ = 1681 ms in women at 3T^51^). ROI_focal_ values were averaged over the four regions per scan for each subject. Individual PWS values of the four hyperintense regions per subject are provided in Supporting Information Figure S2. For the velocity encoding direction, PWS values were normalized to the mean PWS value of all directions to improve visual comparison between subjects. The horizontal bars indicate group means. Post hoc pairwise comparisons *P < .05, **P < .01, ***P < .001. Cutoff velocity = [0.9, 1.6, 4.4, 10.2 cm/s]; Velocity encoding direction = [AP, SI, RL]; PLD = [400, 1000, 1600, 2200 ms]

**FIGURE 8:**
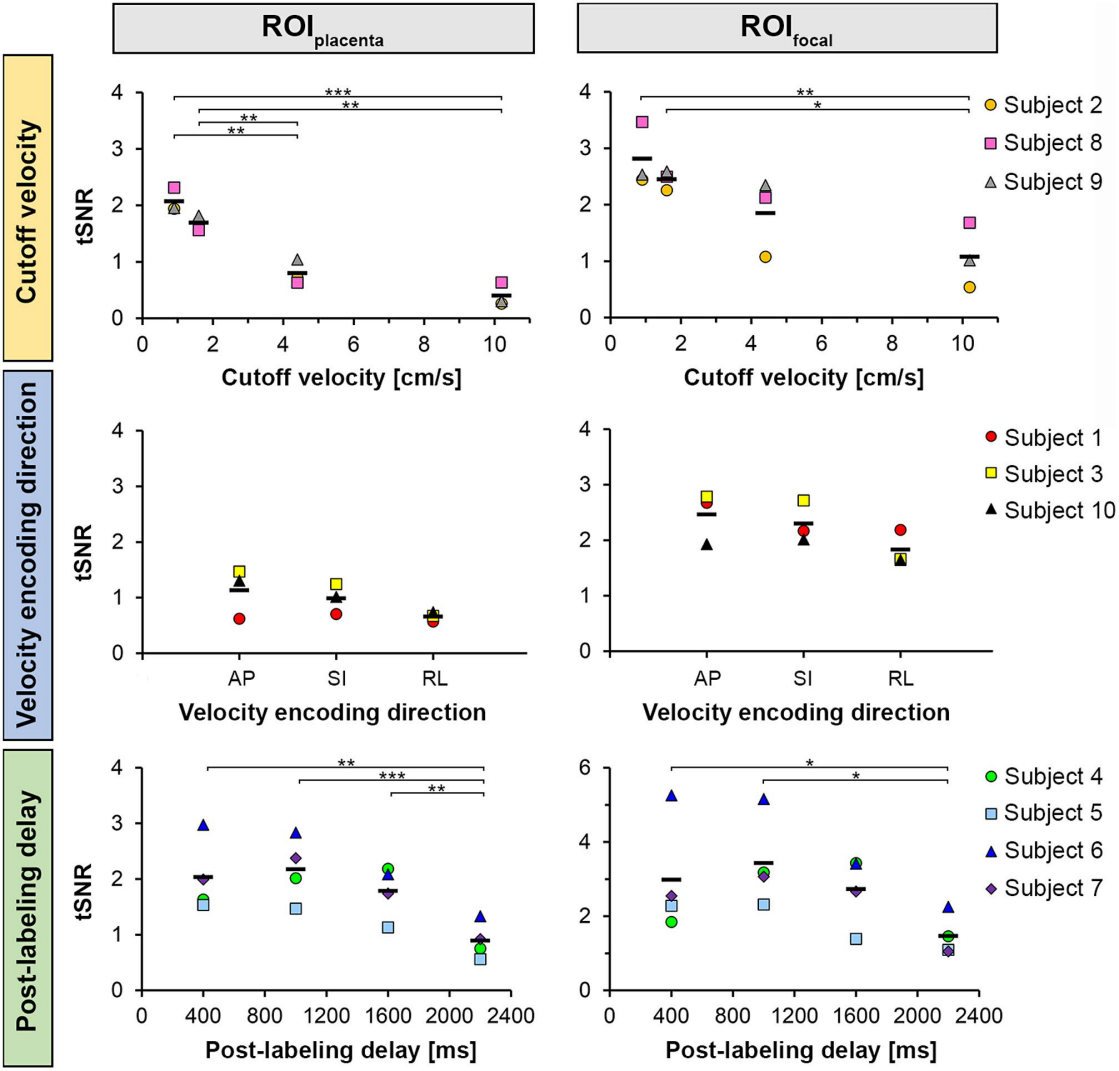
ROI analysis of tSNR obtained from VS‐ASL images with variation of cutoff velocity, velocity encoding direction, and PLD. Data points represent the average PWS in the whole‐placenta region (ROI_placenta_; left column) and in focal hyperintense regions (ROI_focal_; right column). ROI_focal_ values were averaged over the four regions per scan for each subject. Individual tSNR values of the four hyperintense regions per subject are provided in Supporting Information Figure S3. The horizontal bars indicate group means. Post hoc pairwise comparisons *P < .05, **P < .01, ***P < .001. Cutoff velocity = [0.9, 1.6, 4.4, 10.2 cm/s]; Velocity encoding direction = [AP, SI, RL]; PLD = [400, 1000, 1600, 2200 ms]

The perfusion signal intensity decreased with increasing cutoff velocity in all subjects (*n* = 3, GA 28.3‐29.6 weeks); see Figure 6. A diffuse background signal appeared to be visible around the focal hyperintense regions at the lowest cutoff velocity of 0.9 cm/s, for which also diffusion contribution to the VS‐ASL signal was expected to be highest (see Supporting Information Table S1). PWS (Figure 7) and tSNR (Figure 8) decreased with increasing cutoff velocity in all subjects. Whole‐placental PWS showed a 9‐fold decrease over the examined cutoff velocity range (0.9‐10.2 cm/s). There was a statistically significant difference in PWS values measured for both whole‐placenta and focal hyperintense regions between cutoff velocities (*P* < .001). tSNR dropped by almost a factor of 2 in going from a cutoff velocity of 1.6 to 4.4 cm/s. For the focal hyperintense regions the tSNR showed more variation between subjects than the PWS, except for the cutoff velocity of 1.6 cm/s. tSNR values were significantly different between cutoff velocities for both ROIs (*P* < .001 for ROI_placenta_; *P* = .004 for ROI_focal_).

#### 3.3.2 Velocity encoding direction dependence

**FIGURE 9:**
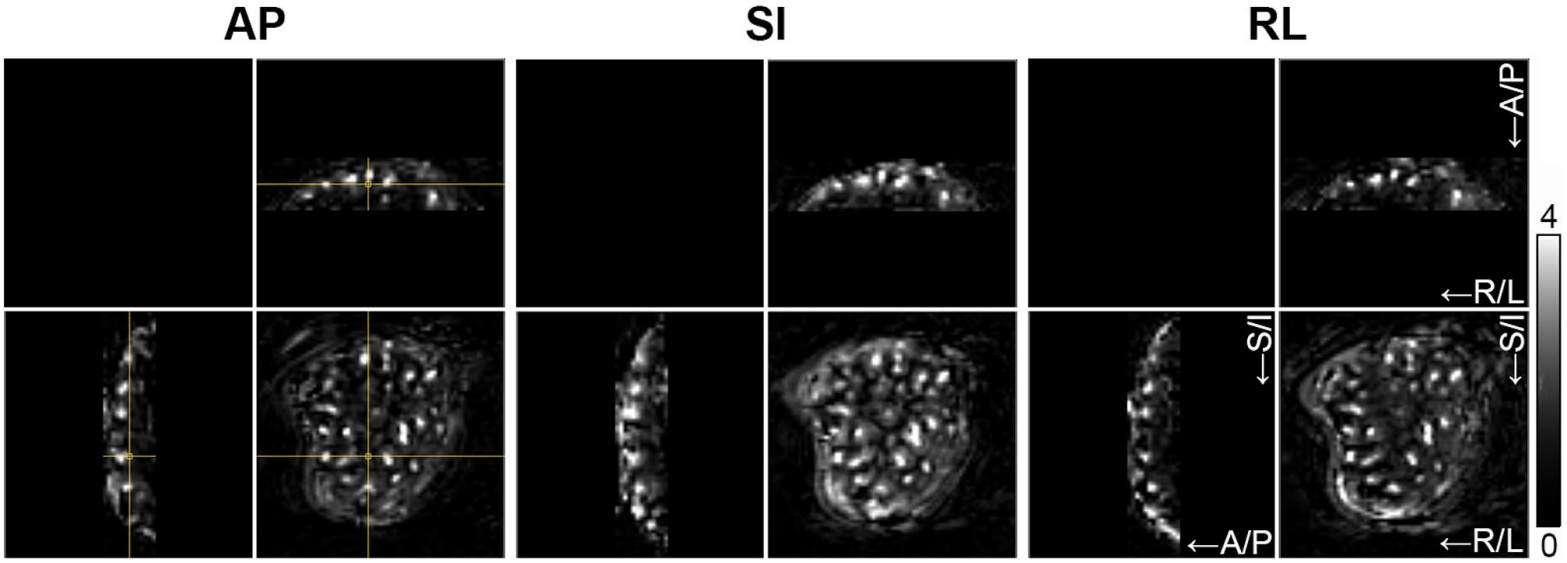
Variation of velocity encoding direction. Placental perfusion images obtained with VS‐ASL MRI using velocity encoding along each of three orthogonal directions (AP, SI, and RL) in one pregnant volunteer (Subject 1 in Table 1). Orthogonal slices (spatial resolution: 4 × 4 × 4 mm^3^) are shown. The cross‐hair in the left images indicates the intersection of the slices. Images have equal LUT scaling. The grayscale bar indicates PWS [%]. A/P, anterior‐posterior; R/L, right‐left; S/I, superior‐inferior

The variation of perfusion signal intensity with velocity encoding direction was not consistent between the three subjects studied (GA 30.0‐35.1 weeks). The example in Figure 9 shows the case of Subject 1 with slightly higher signal intensity with SI‐encoding. Differences in PWS (Figure 7) and tSNR (Figure 8) were observed between encoding directions, but the direction with the highest PWS and tSNR was not the same in all subjects. The normalized voxel‐wise tSD of the motion corrected ASL source images (within ROI_placenta_) was on average 6.3 ± 0.6%, 5.8 ± 1.4%, and 8.5 ± 4.0% for the different encoding directions in Subject 1, 3, and 10, respectively, indicating no consistently higher normalized tSD in any of the directions. PWS and tSNR values were not significantly different between velocity encoding directions in ROI_placenta_ and ROI_focal_ (*P* ≥ .10).

#### 3.3.3 PLD dependence

Overall perfusion signal intensity decreased with increasing PLD in all subjects (*n* = 4 with GA range 24.3‐30.9 weeks); see Figure 3. A more detailed visual evaluation of the perfusion images with increasing PLD indicated that in placental regions close to the fetal side, the PWS increased from PLD 400 to 1000 ms before decreasing again for the longer PLDs (Supporting Information Figure S1). This was observed for all subjects; in one subject (Subject 7), this pattern also occurred in slices closer to the maternal basal plate around the focal hyperintense regions.

Whole‐placenta PWS with PLDs of 400, 1000, and 2200 ms was on average 329 ± 96%, 180 ± 31%, and 47 ± 11% of the PWS measured with the reference PLD of 1600 ms, respectively (Figure 7). The ROI‐averaged PWS decay as a function of PLD appeared to be faster (<1000 ms) than the *T*_1_ decay expected for the *T*_1_ of maternal blood. There was a statistically significant difference in PWS values depending on PLD (*P* < .001 for ROI_placenta_ and ROI_focal_). The highest tSNR was seen at the second PLD of 1000 ms, and the lowest tSNR at the longest PLD in all subjects (Figure 8). tSNR values were significantly different between PLDs for both ROIs (*P* < .001 for ROI_placenta_; *P* = .01 for ROI_focal_). Between subjects, a larger variability was observed for the tSNR values than for the PWS in both ROIs.

#### 3.3.4 Addition of a second VS module before image readout

Placental perfusion images obtained with single and dual VS‐ASL were compared qualitatively, examples are shown in Figure 2. Visually, perfusion signal intensity in the placental parenchyma appeared reduced on the fetal chorionic side with dual VS‐ASL compared with single VS‐ASL, whereas for the focal hyperintense regions the signal intensity appeared similar or increased. Due to the longer TR of dual VS‐ASL, resulting in a longer magnetization recovery time, results between single and dual VS‐ASL were not quantitatively compared.

PWS values from all experiments are provided in Supporting Information Table S2.

## 4 DISCUSSION

The current study focused on the systematic evaluation of essential VS‐ASL sequence parameters to determine optimal settings for placental perfusion measurement. In all subjects, a perfusion pattern with clear hyperintense focal regions was observed. The results show the feasibility of placental perfusion measurements using VS‐ASL with 2D multislice EPI‐readout at 3T MRI, and a clear dependence of perfusion signal on VS labeling parameters and inflow time.

The perfusion patterns observed in this study generally consisted of distinct hyperintense regions spread over the entire placenta, which were more densely located towards the maternal basal plate and surrounded by relatively low signal. The hyperintense regions seem to be part of a normal perfusion pattern in the placenta, and were also observed in other recent placental ASL studies.^19^, ^20^, ^21^ The placenta is a highly vascularized organ containing >60% of blood by volume^28^ (vs 4.5% in the brain) with little tissue/parenchyma for the label to accumulate. Instead, label created on the maternal side accumulates in blood pools inside the IVS, where water and oxygen transfer occurs at the interface with the villous trees; on the fetal side, labeled blood flows to the tips of the villous trees located inside these blood pools, and then turns back into villous venules. Transit time from the spiral artery inlet of the IVS to the uterine vein is estimated to be ~25‐30 s to allow adequate time for O_2_ exchange.^37^ The presence of fetal and maternal blood supply to the placenta adds to the complexity of interpreting the obtained perfusion images. Overall, we expect a larger contribution to the signal from maternal than fetal blood (at GA ~30 weeks relative volumes are roughly 70% vs 30%^38^), assuming equivalent labeling efficiency on maternal and fetal sides.

The PWS in the whole‐placenta ROI with the reference ASL scan showed variation between subjects (0.8‐3.4%). This may be related with the observed negative trend of perfusion signal with increasing GA. Interestingly, the subject with preeclampsia showed the lowest perfusion signal and had one of the longest GA. Thus far, few studies have investigated the relation between perfusion and GA, showing opposite trends in normal^20^, ^39^ and abnormal^19^ pregnancies. Recently, Liu et al showed perfusion‐related image parameters for ischemic placental disease were significantly decreased from normal pregnancy during early gestation.^40^ The relation between perfusion and GA in women with normal and abnormal pregnancies should be further investigated in future studies with larger subject numbers.

As expected, perfusion signal increased with decreasing cutoff velocity. In the current study, we extended the range of cutoff velocities that have been explored by Zun et al,^21^ who evaluated cutoffs of 2 and 4 cm/s. The cutoff velocity determines where in the vascular tree blood is labeled. Maternal blood slows down on entering the IVS, and fetal blood slows down towards the villous tree branches; hence, at lower cutoff velocities a larger fraction of blood is tagged close to the tissue of interest as compared to higher cutoff velocities, and resulting in higher perfusion signal. However, at lower cutoff velocities, the higher gradient amplitudes used in the VS module result in increased contamination by diffusion weighting.^25^, ^27^ Based on the estimated diffusion attenuation of the VS‐ASL signal for each of the used cutoff velocities, it is expected that diffusion weighting had only a minor contribution to the measured perfusion signal, also at the lowest cutoff velocities. In addition, movement of the placenta due to maternal respiration or fetal motion within the range of the cutoff velocity during labeling may influence the apparent perfusion signal measured, due to the tagging of moving tissue. Outlier rejection was performed during post‐processing of the ASL data to remove label‐control pairs with clearly increased signal due to spurious labeling or other artefacts. In the human placenta, flow velocity is typically ≥2 cm/s in spiral artery inlets,^37^, ^41^ ~10‐30 cm/s in the umbilical arteries^42^ and somewhat lower in larger fetal arterioles in the chorionic plate,^43^ and ~8 cm/s in umbilical veins.^44^ The observed dependency of perfusion signal on the applied cutoff velocities indicates that flow velocities in the placental (feeding) vasculature were within this range (0.9‐10.2 cm/s). Blood flow velocities are dependent on pathology, for instance in preeclampsia the input velocity towards the IVS can be much higher,^45^ which may influence labeling and the resulting perfusion signal. Based on our findings, we suggest similar cutoff velocities as used for the reference scan (~1.6 cm/s) since this provided placental ASL images with high perfusion signal, small variations between ASL repetitions, and minimal contamination by diffusion weighting.

The influence of velocity encoding direction on the placental perfusion signal has not been studied yet, and, considering the vasculature feeding the placenta, there is a clear dependence between direction and cutoff velocity. In the brain, the VS‐ASL signal appears to be insensitive to the velocity encoding direction for cutoff velocities below ~4 cm/s, because the cutoff velocity is then low enough to make the method insensitive to geometry of the large arteries.^25^ The placental circulation has a complex vascular structure. Spiral arteries feed the placenta approximately perpendicularly to the basal plate, but the basal plate is in most cases convex. Upstream on the maternal side, the exact shape of the uterine arteries is highly diverse. Fetal blood is flowing from the umbilical arteries towards the chorionic plate in a tortuous path and into the multiple villous trees inside the placenta. In addition, the shape and size of the organ itself and its location within the uterus differs widely (which was partly controlled for in the present study), which may all impact labeling efficiency.

With the assumption that maternal blood is labeled in the spiral arteries close to the inlets into the IVS, it was postulated that perfusion signal would be highest with AP‐encoding for anterior placentas as scanned in this study. However, it was observed that the encoding direction leading to highest perfusion signal varied between subjects. One possible explanation could be that an important part of the label originates from the large feeding vessels that may have substantially different vascular geometries between subjects. To avoid subject‐specific directionality bias, it might be beneficial to perform a combination of velocity encoding directions in one acquisition in an interleaved manner for the acquired label‐control pairs^26^ and then extract a directionally averaged perfusion signal (similarly to diffusion‐weighted trace imaging).

The perfusion signal was highest for the shortest PLD in placental regions close to the maternal basal plate, most likely corresponding to areas close to the inlets of the spiral arteries into the placenta, followed by a near exponential decay. At the shortest PLD on the maternal side of the placenta, the perfusion signal is first mainly dominated by high signal, most likely due to labeled blood that is present in the larger vessels before entering the IVS of the placenta. This large vascular contribution to the perfusion signal at the shortest PLD may explain the *T*_1_ decay that is apparently faster than expected. In placental tissue closer to the fetal side, the signal appeared instead to peak at the second PLD (1300‐1700 ms depending on slice number) indicating a slightly delayed arrival of labeled blood. This is consistent with the labeled blood arriving in the depths of the IVS or possibly labeled water^46^ being transferred to the fetal blood at the interface of the villous trees. It would be interesting to perform additional measurements with shorter, longer and more closely spaced PLD values to better characterize blood flow dynamics in placental regions across the fetal‐maternal axis.

As a consequence of on one hand linear accumulation of label with PLD because of flow and on the other hand the *T*_1_ exponential decay, perfusion signal in tissue (eg, cerebral gray matter) often has an optimum at PLD~*T*_1_. This value is also dependent on tagging location and, thus, cutoff velocity. In the placenta, it may be advantageous to decrease this parameter to ~1000 ms to obtain higher tSNR with good image contrast on the perfusion images, according to our data at a cutoff velocity of 1.6 cm/s. Most importantly however, rather than using the PLD to optimize tSNR and PWS, the PLD determines label distribution in the placenta. Measurement at multiple PLDs may be of benefit in revealing differences between healthy and diseased placental blood flow, such as disruption of blood flow within the IVS.

In the brain, typically a second VS module is used before the image readout to reduce venous signal contributions and to allow quantification. Since the placental circulation is radically different from the brain, the current study primarily focused on measuring the distribution of labeled blood in the placenta using a single VS module, without complicating the measurement with the second VS module. However, visual comparison of single and dual VS‐ASL contrast in the placenta may help to understand which parts of the circulation are included in the measurement. The contrast between focal hyperintense regions and placenta parenchyma increased for dual VS‐ASL as compared to single VS‐ASL. This would be consistent with the hypothesis that the observed hyperintense regions represent labeled blood accumulating in the IVS, as labeled blood inside the IVS has slow, non‐laminar flow and would therefore be hardly affected by the second VS module, as opposed to vascular flow in the parenchyma. Within the placental parenchyma, the perfusion signal intensity difference between maternal and fetal side appeared slightly increased with dual VS‐ASL. A possible explanation would be saturation of the label in the dense vasculature on the fetal side as its velocity exceeds the cutoff velocity. Alternatively, the *T*_2_‐related attenuation of the tagged magnetization during the second VS module could be stronger on the fetal side due to a shorter *T*_2_ of fetal blood (assuming reduced oxygenation levels compared with the mother).^47^

Based on the results of this study, cutoff velocity similar as used for the brain (~1.6 cm/s) seems optimal for the placenta. At this cutoff velocity, relatively shorter inflow times ~1000 ms can be applied to obtain good image contrast on the perfusion images with high tSNR, although multiple PLDs may also allow characterization of blood flow dynamics across the fetal‐maternal axis. A combination of velocity encoding directions in one acquisition could be beneficial to avoid individual directionality effects. The proposed parameter settings provide a basis for setting up an efficient VS‐ASL protocol, which could be used to investigate the natural course of placental perfusion, and make first observations of differences with pathology. Future studies can expand on the presented technique, further probing the underlying placental physiology by measuring multiple VS‐ASL parameter combinations. There is also great potential in combining VS‐ASL with other contrast mechanisms, such as $T_{2}^{*}$ measurements to detect blood oxygenation^48^, ^49^ or angiography to better visualize placental vasculature.

This study has limitations. First, each sequence parameter was explored in a small number of subjects. This is largely related to the limited time available per subject (~20 min) and also meant that repeatability could not be investigated within this study. In general it is challenging to perform an extensive scan protocol in a study population with pregnant subjects. Based on the current results, we can explore specific parameter combinations instead of only one parameter per subject. Second, only anterior located placentas were included. Third, imaging occurred relatively late in gestation while the assessment of placental function would be most useful in earlier stages of pregnancy. Finally, the used VS preparation method is susceptible to eddy currents that could result in subtraction artefacts at short PLDs even with a minimum PLD of 400 ms. Using a different VS preparation method that is more robust to eddy currents (eg, BIR‐8) may enable acquisitions at even shorter PLDs.^33^, ^50^

## 5 CONCLUSIONS

The results presented confirm the feasibility of using VS‐ASL with 2D multislice readout at 3T to generate perfusion‐weighted signal in the placenta, and indicate a clear dependence of placental perfusion signal on VS labeling and inflow time (PLD) settings. Based on our findings a practical and effective VS‐ASL protocol to measure placental perfusion can be defined, pointing the way towards an additional noninvasive tool to characterize an important placenta functional measure, which could be applied alongside ultrasound and other existing MRI methods to characterize both normal placental development and pathology.

## Supporting information


**FIGURE S1** Placental perfusion images obtained with single VS‐ASL MRI using four different post‐labeling delays (400, 1000, 1600, and 2200ms) in one pregnant volunteer (Subject 4 in Table 1). Slices with maternal coronal (rows 1‐2), axial (rows 3‐4; red line), and sagittal (rows 5‐6; blue line) orientation (spatial resolution: 4 × 4 × 4 mm^3^) are shown. The yellow asterisk on the axial/sagittal slices points at the fetal chorionic side of the placenta. Perfusion images are shown with optimal LUT scaling for each individual post‐labeling delay (rows 1, 3, 5) and equal scaling between all delay times (rows 2, 4, 6). Dynamics graphs of the signal at multiple post‐labeling delays are shown that were measured at multiple locations on the fetal (yellow crosses) and maternal side (blue crosses) of the placenta
**FIGURE S2** Individual PWS values of the four hyperintense regions per subject obtained from VS‐ASL images with variation of cutoff velocity (left column), velocity encoding direction (middle column), and post‐labeling delay (right column). Each data point represents the PWS averaged over all voxels inside one focal hyperintense region (ROI_focal_). The red horizontal bars indicate group means. Cutoff velocity = [0.9, 1.6, 4.4, 10.2 cm/s]; Velocity encoding direction = [AP, SI, RL]; Post‐labeling delay = [400, 1000, 1600, 2200 ms]
**FIGURE S3** Individual tSNR values of the four hyperintense regions per subject obtained from VS‐ASL images with variation of cutoff velocity (left column), velocity encoding direction (middle column), and post‐labeling delay (right column). Each data point represents the voxel‐wise tSNR averaged over all voxels inside one focal hyperintense region (ROI_focal_). The red horizontal bars indicate group means. Cutoff velocity = [0.9, 1.6, 4.4, 10.2 cm/s]; Velocity encoding direction = [AP, SI, RL]; Post‐labeling delay = [400, 1000, 1600, 2200 ms]
**TABLE S1** Overview of the applied VS module settings for each cutoff velocity with the resulting *b*‐values and estimated subtraction error due to diffusion during the VS module. The cutoff velocity was varied by changing the gradient strength
**TABLE S2** Overview of PWS values measured with the different parameter settings in each subject
