# Supplementary material for: Systematic evaluation of velocity‐selective arterial spin labeling settings for placental perfusion measurement
Source: Magn Reson Med. 2020 Mar 6;84(4):1828–43. doi: 10.1002/mrm.28240 (PMC7384055; doi:10.1002/mrm.28240)
Supplement: Supplementary file 1 — FIGURE S1 Placental perfusion images obtained with single VS‐ASL MRI using four different post‐labeling delays (400, 1000, 1600, and 2200ms) in one pregnant volunteer (Subject 4 in Table 1). Slices with maternal coronal (rows 1‐2), axial (rows 3‐4; red line), and sagittal (rows 5‐6; blue line) orientation (spatial resolution: 4 × 4 × 4 mm3) are shown. The yellow asterisk on the axial/sagittal slices points at the fetal chorionic side of the placenta. Perfusion images are shown with optimal LUT scaling for each individual post‐labeling delay (rows 1, 3, 5) and equal scaling between all delay times (rows 2, 4, 6). Dynamics graphs of the signal at multiple post‐labeling delays are shown that were measured at multiple locations on the fetal (yellow crosses) and maternal side (blue crosses) of the placenta FIGURE S2 Individual PWS values of the four hyperintense regions per subject obtained from VS‐ASL images with variation of cutoff velocity (left column), velocity encoding direction (middle column), and post‐labeling delay (right column). Each data point represents the PWS averaged over all voxels inside one focal hyperintense region (ROIfocal). The red horizontal bars indicate group means. Cutoff velocity = [0.9, 1.6, 4.4, 10.2 cm/s]; Velocity encoding direction = [AP, SI, RL]; Post‐labeling delay = [400, 1000, 1600, 2200 ms] FIGURE S3 Individual tSNR values of the four hyperintense regions per subject obtained from VS‐ASL images with variation of cutoff velocity (left column), velocity encoding direction (middle column), and post‐labeling delay (right column). Each data point represents the voxel‐wise tSNR averaged over all voxels inside one focal hyperintense region (ROIfocal). The red horizontal bars indicate group means. Cutoff velocity = [0.9, 1.6, 4.4, 10.2 cm/s]; Velocity encoding direction = [AP, SI, RL]; Post‐labeling delay = [400, 1000, 1600, 2200 ms] TABLE S1 Overview of the applied VS module settings for each cutoff velocity with the resulting b‐values and [file MRM-84-1828-s001.pdf]

Supporting Information

Systematic evaluation of velocity-selective arterial spin labeling settings for placental perfusion measurement

Magnetic Resonance in Medicine

Anita A. Harteveld, PhD[1], Jana Hutter, PhD[2], Suzanne L. Franklin, MSc[1,3], Laurence H. Jackson, PhD[2], Mary Rutherford, MD PhD[2], Joseph V. Hajnal, PhD[2], Matthias J.P. van Osch, PhD[3], Clemens Bos, PhD[1], Enrico De Vita, PhD[2]

[1]Department of Radiology, University Medical Center Utrecht, Utrecht University, Utrecht, the Netherlands
[2]Biomedical Engineering, School of Imaging Sciences and Biomedical Engineering, King's College London, London, United Kingdom
[3]C.J. Gorter Center for high field MRI, Department of Radiology, Leiden University Medical Center, Leiden, the Netherlands

E-mail address corresponding author: a.a.harteveld-2@umcutrecht.nl

Figures

Supporting Information Figure S1. Placental perfusion images obtained with single VS-ASL MRI using four different post-labeling delays (400, 1000, 1600 and 2200ms) in one pregnant volunteer (Subject 4 in Table 1). Slices with maternal coronal (rows 1-2), axial (rows 3-4; red line), and sagittal (rows 5-6; blue line) orientation (spatial resolution: 4x4x4 mm^3) are shown. The yellow asterisk on the axial/sagittal slices points at the fetal chorionic side of the placenta. Perfusion images are shown with optimal LUT scaling for each individual post-labeling delay (rows 1, 3, 5) and equal scaling between all delay times (rows 2, 4, 6). Dynamics graphs of the signal at multiple post-labeling delays are shown that were measured at multiple locations on the fetal (yellow crosses) and maternal side (blue crosses) of the placenta.

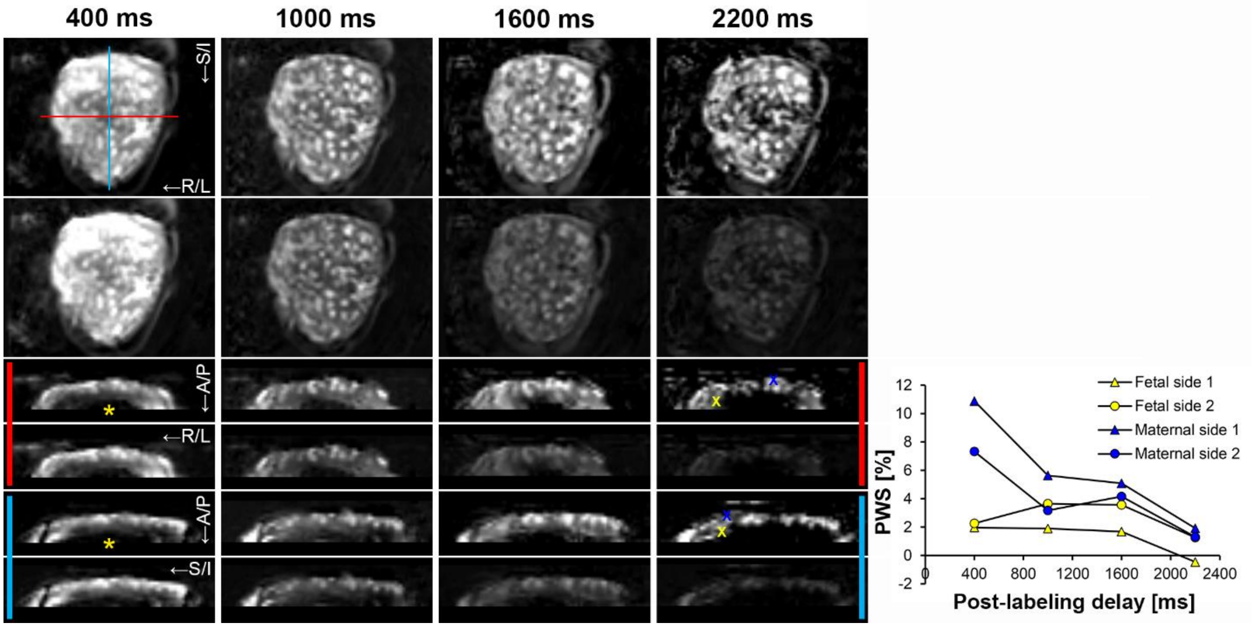

Supporting Information Figure S2. Individual PWS values of the four hyper-intense regions per subject obtained from VS-ASL images with variation of cutoff velocity (left column), velocity encoding direction (middle column), and post-labeling delay (right column). Each data point represents the PWS averaged over all voxels inside one focal hyper-intense region (ROI_{focal}). The red horizontal bars indicate group means. *Cutoff velocity = [0.9, 1.6, 4.4, 10.2cm/s]; Velocity encoding direction = [AP, SI, RL]; Post-labeling delay = [400, 1000, 1600, 2200ms].*

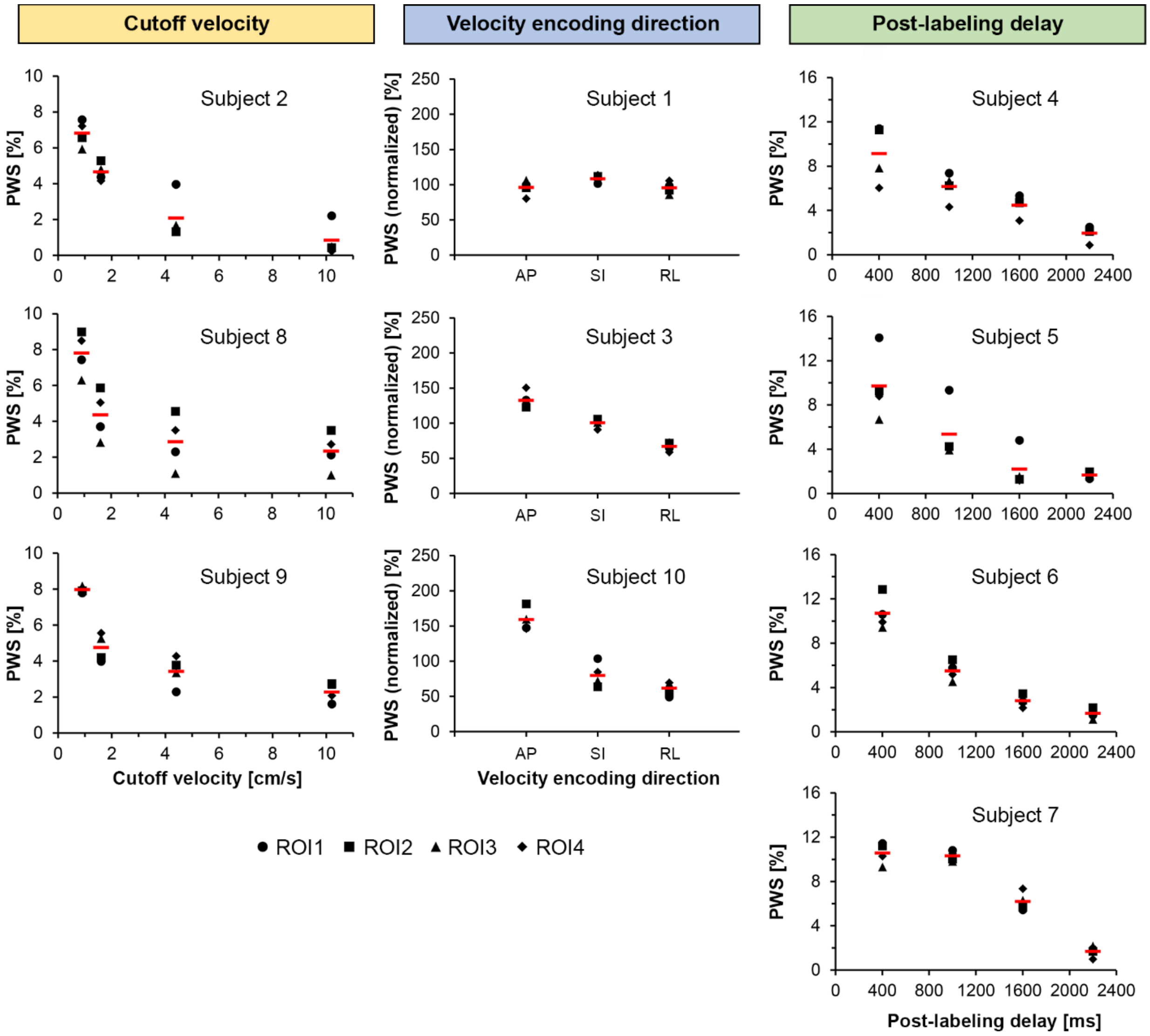

Supporting Information Figure S3. Individual tSNR values of the four hyper-intense regions per subject obtained from VS-ASL images with variation of cutoff velocity (left column), velocity encoding direction (middle column), and post-labeling delay (right column). Each data point represents the voxel-wise tSNR averaged over all voxels inside one focal hyper-intense region (ROI_{focal}). The red horizontal bars indicate group means. *Cutoff velocity = [0.9, 1.6, 4.4, 10.2cm/s]; Velocity encoding direction = [AP, SI, RL]; Post-labeling delay = [400, 1000, 1600, 2200ms].*

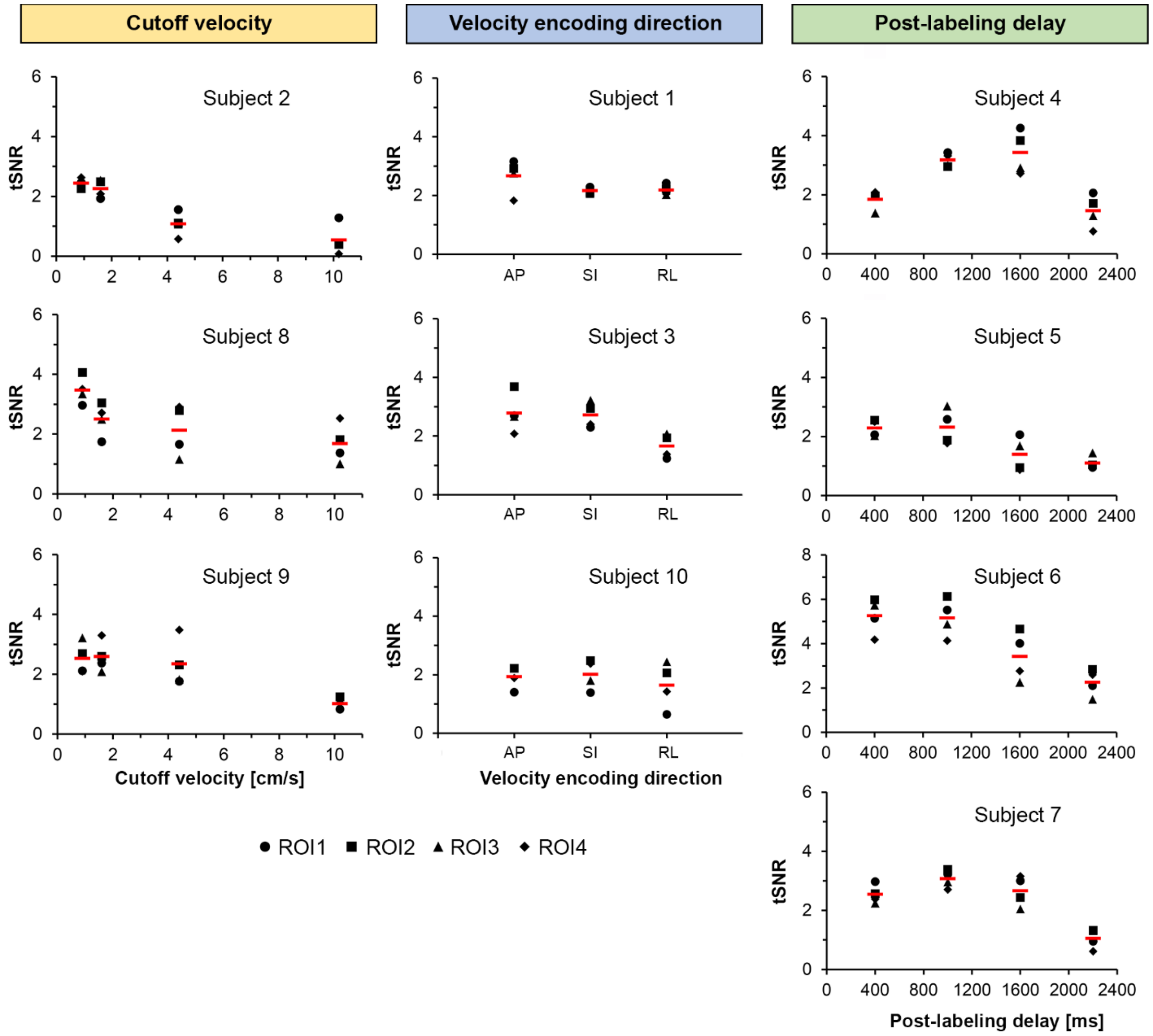

Tables

Supporting Information Table S1. Overview of the applied VS module settings for each cutoff velocity with the resulting b-values and estimated subtraction error due to diffusion during the VS module. The cutoff velocity was varied by changing the gradient strength.

Cutoff velocity [cm/s][a]	T_{VS} [ms]	G [mT/m]	δ [ms][b]	Δ [ms]	Δ' [ms]	b-value [s/mm^2]	1-exp(-b/ADC) [%, placenta][c]
0.9	50	19	1.43	47.2	2.16	2.47	0.443
1.6	50	13	1.20	46.8	1.70	0.81	0.145
4.4	50	6	0.93	46.2	1.16	0.10	0.018
10.2	50	3	0.82	46.0	0.93	0.02	0.004

[a]Cutoff velocity was calculated using: $V_c = \pi / (\gamma \cdot G \cdot \delta \cdot (\Delta + \Delta'))$.
[b]The duration of the gradient plateau remained constant at 0.7ms for each gradient strength.
[c]Upper limits of the estimated diffusion contribution to the VS-ASL signal at PLD=0 s. For PLD>0 s T_1 decay over time should be taken into account as exp(-PLD/$T_{1,tissue}$). Expected diffusion attenuation of the VSASL signal was estimated with 1-exp(-b·ADC)[1] using the b-value from the applied VS-gradients ($b = \gamma^2 \cdot G^2 \cdot \delta^2 \cdot (\Delta - \delta / 3)$) and the tissue apparent diffusion coefficient ($ADC_{placenta}=1.8\cdot10^{-3}$ mm^2/s [2, 3]).

T_{VS}: duration VS module; G: gradient strength; δ: duration gradient plateau + one slope; Δ: duration from the middle of the first gradient to the middle of the fourth gradient; Δ': duration from the middle of the second gradient to the middle of the third gradient.

Supporting Information Table S2. Overview of PWS values measured with the different parameter settings in each subject.

Subject	Experiment[a]	ROI[b]									
		placenta		focal1		focal2		focal3		focal4	
		mean	*SD*	*mean*	*SD*	*mean*	*SD*	*mean*	*SD*	*mean*	*SD*
1	**V1**	0.8	0.9	4.1	1.8	3.1	1.4	4.0	1.9	2.7	1.1
	V2	1.2	1.1	4.2	1.5	3.7	1.1	4.0	1.3	3.8	1.0
	V3	0.8	0.9	4.0	1.8	3.0	1.3	3.2	1.5	3.5	1.3
2	C1	5.2	1.9	7.6	2.2	6.6	0.3	5.9	1.6	7.2	1.4
	**C2**	3.3	1.6	4.4	1.5	5.3	1.8	4.8	1.5	4.2	1.2
	C3	1.1	1.0	4.0	1.6	1.3	0.4	1.7	1.4	1.3	0.5
	C4	0.4	0.9	2.2	1.2	0.4	0.5	0.5	0.8	0.2	0.3
	Dual	5.2	2.3	5.6	1.1	5.8	1.4	6.0	1.7	4.8	0.8
3	**V1**	2.7	1.8	4.8	1.2	4.7	1.3	3.4	0.8	4.2	1.2
	V2	1.8	1.3	3.7	1.5	4.0	1.3	2.7	0.9	2.5	0.9
	V3	1.0	1.3	2.3	1.2	2.7	1.1	2.0	1.2	1.6	1.1
4	PLD1	7.2	2.9	11.4	1.8	11.3	1.8	7.8	1.1	6.0	1.3
	PLD2	3.8	1.6	7.3	1.6	6.2	1.9	6.6	1.2	4.3	1.5
	**PLD3**	2.8	1.0	5.3	1.4	4.7	1.6	4.7	1.1	3.1	0.8
	PLD4	1.1	0.7	2.5	0.6	2.1	0.7	2.2	0.4	0.9	0.5
	Dual	2.8	1.2	5.1	1.2	5.3	1.0	3.9	0.8	2.4	1.2
5	PLD1	6.0	3.3	14.1	8.5	9.3	3.1	6.7	2.7	8.8	3.1
	PLD2	2.9	2.1	9.3	5.6	4.3	1.1	3.9	2.0	4.0	1.2
	**PLD3**	1.3	1.1	4.8	1.8	1.3	0.7	1.5	0.3	1.2	0.6
	PLD4	0.8	0.8	1.4	0.5	2.0	0.4	1.6	0.7	1.8	0.6
	Dual	1.6	1.3	4.2	1.4	4.5	1.7	2.6	1.2	4.1	1.8
6	PLD1	8.0	3.5	10.6	2.4	12.9	4.0	9.4	1.8	9.9	2.5
	PLD2	3.7	1.6	5.8	1.5	6.5	1.7	4.5	1.2	5.2	1.8
	**PLD3**	2.2	0.9	2.7	0.3	3.4	0.4	2.9	0.7	2.2	0.5
	PLD4	1.2	0.7	1.5	0.6	2.2	0.5	1.1	0.4	1.8	0.6
	Dual	2.3	0.9	3.0	0.7	3.5	0.3	2.8	0.8	3.6	0.9
7	PLD1	7.6	2.9	11.4	3.0	11.2	3.4	9.3	2.5	10.3	2.5
	PLD2	6.6	2.2	10.8	3.1	10.0	1.8	9.8	1.6	10.5	2.2
	**PLD3**	3.4	2.1	5.4	1.4	5.7	1.2	6.3	1.4	7.3	1.9
	PLD4	1.2	0.8	1.9	0.4	1.7	1.1	2.2	0.8	1.0	0.5
	Dual	4.4	1.9	6.6	1.8	4.5	0.5	5.8	0.5	6.5	0.7
8	C1	4.7	2.8	7.4	1.0	9.0	2.5	6.3	0.9	8.5	0.9
	**C2**	2.2	1.6	3.7	1.5	5.9	2.6	2.8	0.7	5.0	1.1
	C3	0.8	1.1	2.3	1.5	4.6	2.8	1.1	0.6	3.5	1.2
	C4	0.7	1.0	2.1	1.2	3.5	2.2	1.0	0.4	2.7	1.4
	Dual	2.0	2.1	4.7	2.6	4.9	2.5	2.0	2.1	3.4	1.8
9	C1	4.3	2.1	7.8	0.8	7.9	0.6	8.2	1.0	8.0	1.0
	**C2**	2.4	1.2	4.0	1.6	4.2	1.4	5.3	1.2	5.6	1.5
	C3	1.2	1.0	2.3	1.6	3.8	1.5	3.3	1.7	4.3	1.7
	C4	0.5	0.7	1.6	1.0	2.7	1.1	2.7	1.0	2.1	1.4

10	**V1**	2.8	1.7	4.5	1.7	6.5	1.4	6.7	0.8	5.4	1.5
	V2	1.2	1.0	3.2	1.5	2.3	1.3	3.0	1.3	3.1	1.5
	V3	1.0	1.0	1.5	1.3	2.0	1.2	2.9	0.8	2.6	1.7

[a]The reference VS-ASL scan is highlighted in **bold**.

[b]Mean and SD values [%] are provided for the whole-placenta region ($ROI_{placenta}$) and the four focal hyper-intense regions (ROI_{focal}).

Dual: dual velocity-selective arterial spin labeling; C1: cutoff velocity 0.9 cm/s; C2: cutoff velocity 1.6 cm/s; C3: cutoff velocity 4.4 cm/s; C4: cutoff velocity 10.2 cm/s; PLD1: post-labeling delay 400 ms; PLD2: post-labeling delay 1000 ms; PLD3: post-labeling delay 1600 ms; PLD4: post-labeling delay 2200 ms; V1: velocity encoding direction anterior-posterior; V2: velocity encoding direction superior-inferior; V3: velocity encoding direction right-left

References

1. Schmid S, Ghariq E, Teeuwisse WM, Webb A, van Osch MJ. Acceleration-selective arterial spin labeling. Magn Reson Med. 2014;71:191-199
2. Shapira-Zaltsberg G, Grynspan D, Reddy D, Miller E. Apparent Diffusion Coefficient of the Placenta in Twin versus Singleton Pregnancies. Fetal Diagn Ther 2018;44:129-134
3. Siauve N, Chalouhi GE, Deloison B, Alison M, Clement O, Ville Y, et al. Functional imaging of the human placenta with magnetic resonance. Am J Obstet Gynecol. 2015;213:S103-114
